# HAITCH: A framework for distortion and motion correction in fetal multi-shell diffusion-weighted MRI

**DOI:** 10.1162/imag_a_00490

**Published:** 2025-02-26

**Authors:** Haykel Snoussi, Davood Karimi, Onur Afacan, Mustafa Utkur, Ali Gholipour

**Affiliations:** Department of Radiology, Boston Children’s Hospital, and Harvard Medical School, Boston, MA, United States; Department of Radiological Sciences, University of California Irvine, Irvine, CA, United States; Department of Electrical Engineering and Computer Science, University of California Irvine, Irvine, CA, United States

**Keywords:** fetal imaging, diffusion MRI, multi-shell, motion correction, dynamic distortion correction

## Abstract

Diffusion magnetic resonance imaging (dMRI) is pivotal for probing the microstructure of the rapidly-developing fetal brain. However, fetal motion during scans and its interaction with magnetic field inhomogeneities result in artifacts and data scattering across spatial and angular domains. The effects of those artifacts are more pronounced in high-angular resolution fetal dMRI, where signal-to-noise ratio is very low. Those effects lead to biased estimates and compromise the consistency and reliability of dMRI analysis. This work presents High Angular resolution diffusion Imaging reconsTruction and Correction approacH (HAITCH), the first and the only publicly available tool to correct and reconstruct multi-shell high-angular resolution fetal dMRI data. HAITCH offers several technical advances that include a blip-reversed dual-echo acquisition for dynamic distortion correction, advanced motion correction for*model-free*and robust reconstruction, optimized multi-shell design for enhanced information capture and increased tolerance to motion, and outlier detection for improved reconstruction fidelity. The framework is open-source, flexible, and can be used to process any type of fetal dMRI data, including single-echo or single-shell acquisitions, but is most effective when used with multi-shell multi-echo fetal dMRI data that cannot be processed with any of the existing tools. Validation experiments on real fetal dMRI scans demonstrate significant improvements and accurate correction across diverse fetal ages and motion levels. HAITCH successfully removes artifacts and reconstructs high-fidelity fetal dMRI data suitable for advanced diffusion modeling, including fiber orientation distribution function estimation. These advancements pave the way for more reliable analysis of the fetal brain microstructure and tractography under challenging imaging conditions.

## 1 Introduction

Fetal diffusion magnetic resonance imaging (dMRI) provides detailed insights into the development of brain connectivity and microstructure during the prenatal period (Calixto, Machado-Rivas, et al., 2024;Calixto, Soldatelli, et al., 2024;Calixto, Taymourtash, et al., 2024;Calixto et al., 2023;Deprez et al., 2020;Hunt et al., 2019;Hutter et al., 2018;Jaimes et al., 2020;Jakab, 2019;Jakab et al., 2017;Karimi et al., 2024;Kebiri et al., 2024;Khan et al., 2019;Machado-Rivas et al., 2021). This powerful technique relies on a mathematical model that delves into the microscopic behavior of water molecules within brain tissue. This model begins by calculating the probability$P({\text{R}}_{\tau }|{\text{R}}_{\text{0}},\tau )$, which represents the likelihood of a water molecule moving from position${\text{R}}_{\text{0}}$to${\text{R}}_{\tau }$over timeτ. Since it is impossible to determine this probability for a single molecule, we calculate the Ensemble Average Propagator (EAP),P(R), which represents the averaged probability across all molecules within a voxel. Building upon Stejskal and Tanner’s work (Stejskal & Tanner, 1965on the pulsed gradient spin-echo sequence), we can express the normalized dMRI signal,E(q), as the Fourier transform of the EAP. When the diffusion-weighted gradient durationδis sufficiently shorter than the time between two gradient pulsesΔ,E(q)can be expressed as:

$$E\left(\text{q}\right)=\int_{R\in {\mathbb{R}}^{3}}\;P\left(\text{R}\right)\;\text{exp}\left(-2\pi i\text{q}\cdot \text{R}\right)\;d\text{R}$$ (1)

whereqis a 3D-vector representing the effective gradient direction (q=quwhereuis a 3D unit vector).

A standard dMRI scan consists of acquiring a reference insensitive to diffusion (b-value=0) and a set of diffusion-weighted images (b-value>0) in non-collinear directions. By employing a suitable diffusion model, this setup allows the estimation of the local diffusion properties and the brain’s structural connectivity network. The diffusion model estimation assumes that the gathered dMRI signals originate from the same physical point. Achieving this condition in fetal scans, however, is challenging due to several factors, such as maternal breathing and unpredictable fetal motion (Deprez et al., 2020;Gholipour et al., 2014). These issues cause misalignment in dMRI sequences and disrupt the assumption of a consistent relationship between image space and anatomy. Moreover, the inherent limitations of echo planar imaging (EPI) used in dMRI acquisition, such as geometric distortion and susceptibility to spin history effects, distort the dMRI signals (Afacan et al., 2019;Andersson, 2021;Hutter et al., 2018;Snoussi et al., 2019,^2021^). The problem is magnified in the context of fetal imaging due to the complex interaction between fetal motion and the susceptibility-induced inhomogeneities of the main magnetic field (B0), combined with the small size of the fetal brain and the low signal-to-noise ratio (SNR). Inconsistencies originating from all these factors are a major impediment to accurately characterize fetal brain connectivity and hinder the overall reproducibility of*in-utero*dMRI studies (Jakab et al., 2017;Xiao et al., 2024).

To address these challenges, several fetal-specific retrospective motion correction methods have been developed. The initial approach of motion correction for fetal dMRI, introduced byJiang et al. (2009), extended slice-to-volume reconstruction (SVR) techniques originally developed for*in-utero*structural MRI. By assuming that a rank-2 tensor model can represent local diffusion properties,Jiang et al. (2009)registered each slice to the simulated volume. A diffusion tensor matrix reconstruction was then employed to integrate the realigned slices into a regular grid to produce the final reconstructed volume.Oubel et al. (2012)proposed a groupwise registration method that aligns diffusion-weighted images collectively before utilizing a derived image for registration with the T2-weighted (T2w) reference image. Affine transformation matrices were applied to realign the original sequences, addressing both motion and eddy-current effects. Then, a dual radial basis function-based interpolation was used to reconstruct a consistent image.Fogtmann et al. (2013)presented a super-resolution reconstruction of the diffusion tensors, with some similarities in the registration concept toOubel et al. (2012)and several key distinctions fromScherrer et al. (2012). Notable differences include the implementation of a unified reconstruction-alignment formulation, yielding a diffusion-sensitive slice registration model. Additionally, Fogtmann et al. incorporated point spread function deconvolution into the 3D image reconstruction process. This allowed the creation of 3D datasets with isotropic spatial resolution from multiple scattered slices acquired in different anatomical planes.Marami et al. (2017)proposed motion correction and reconstruction through a dynamic model of fetal head motion; where motion parameters are estimated through a Kalman filtering approach. Additionally, they reconstructed diffusion tensors using a weighted least-squares fit. This approach notably contributed to the creation of the first spatio-temporal diffusion tensor fetal atlas, as detailed inKhan et al. (2019).Deprez et al. (2020)advanced the field by correcting motion using super-resolution reconstruction with spherical harmonics (SH). This approach is particularly effective in identifying crossing fibers in the fetal brain. Building upon their previous work (Deprez et al., 2012), the authors notably included intensity correction within the reconstruction process.

The above-mentioned techniques have predominantly employed data representations grounded in diffusion tensor imaging and single-shell SH. However, these approaches have limitations in capturing the complexity of the developing brain. The tensor model struggles to represent crossing fibers which is a prevalent feature in fetal brain white matter. A*model-free*signal representation is crucial for such motion correction methodologies to guarantee a broad range of imaging analyses. Similarly,Tournier et al. (2020)highlight the insufficiency of single-shell SH in capturing the full spectrum of diffusion information needed for neonatal imaging, which is highly similar to fetal imaging. Consequently, these methodologies either restrict the type of diffusion information extracted or reduce the information content and impose constraints on the variety of input data that can be processed. This significantly hinders the scope of subsequent analytical endeavors aimed at comprehensively characterizing the developing fetal brain microstructure. Moreover, the effect of local geometric distortions and their interaction with fetal and maternal motion has been either ignored or not adequately addressed in those studies. While conventional methods for EPI distortion correction rely on static field mapping (Jezzard & Balaban, 1995), which is only appropriate for motion-free data, methods that rely on dMRI volumes with reversed phase encoding are not effective for the continuous and large motion that typically affects fetal dMRI (Andersson et al., 2003,^2017^). This warrants further investigation of dMRI acquisition and processing methods to address motion and geometric distortions.

In this work, we introduce the High Angular resolution diffusion Imaging reconsTruction and Correction approacH (HAITCH), a novel framework that tackles these identified challenges by integrating optimized acquisition and reconstruction strategies to mitigate the combined effects of fetal motion, geometric distortion, and their interaction in fetal dMRI. To this end, our objective was to develop a robust methodology and a toolbox for acquiring and reconstructing fetal dMRI data that ensures high fidelity and suitability for detailed*in-vivo*and*in-utero*analysis of the fetal brain microstructure development.

To achieve this, HAITCH focuses on three core technical contributions, represented by the bold-bordered boxes inFigure 1:*i)*Optimized multi-shell sampling scheme for improved fetal dMRI,*ii)*Dual-Echo sequence enables dynamic distortion correction, and*iii)*Advanced motion correction and model-free reconstruction techniques. We validate the accuracy of our framework using real fetal dMRI scans acquired at Boston Children’s Hospital. The implementation of the HAITCH framework is publicly available as the first module of the Fetal and Neonatal Development Imaging (FEDI) toolbox:https://fedi.readthedocs.io.

**Fig. 1.**
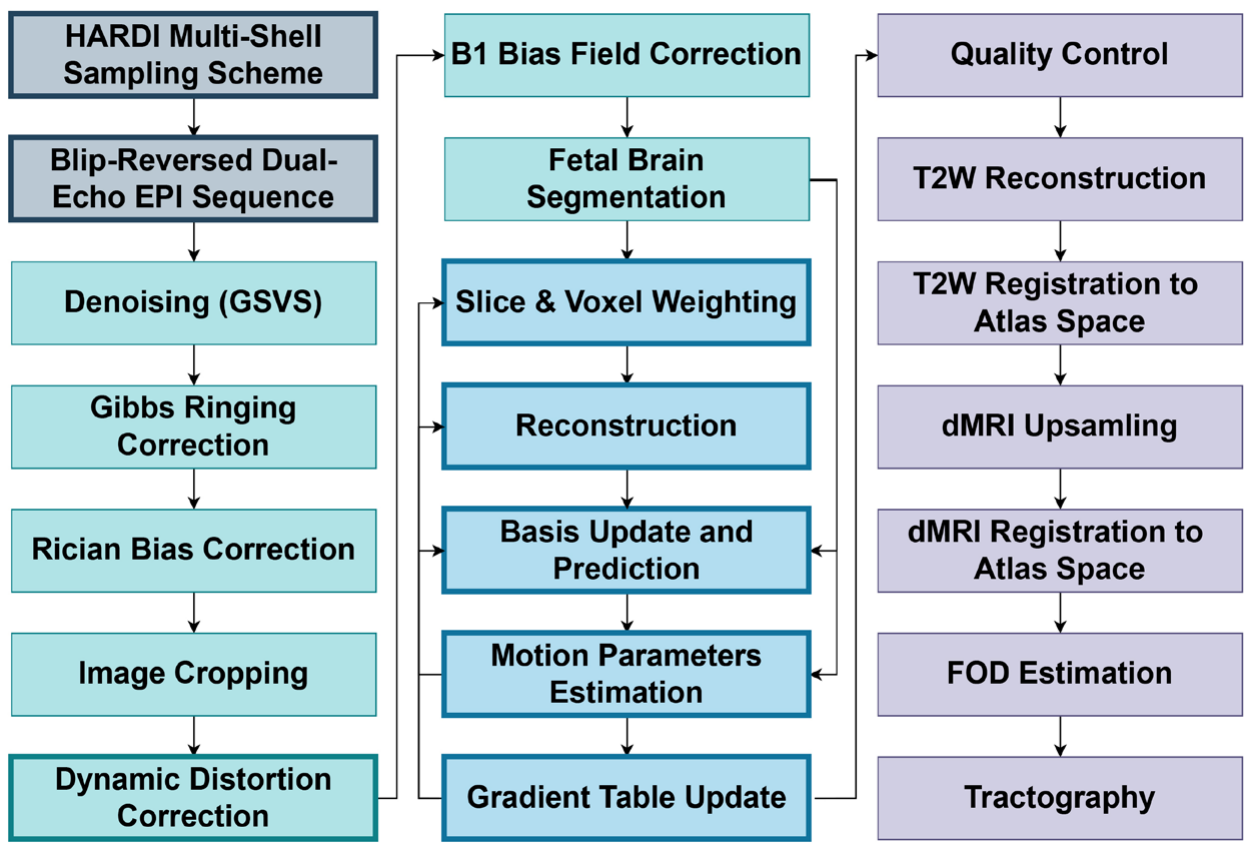
HAITCH Framework Processing Stages. This flowchart illustrates the key steps involved in the HAITCH Framework. Grey boxes outline the acquisition stages, including the sampling scheme and the dual-echo sequence. Green boxes detail the pre-processing steps, distortion correction, B1 bias field correction, and fetal brain segmentation. Blue boxes refer to the iterative approach of motion correction and image reconstruction that includes slice & voxel weighting,*model-free*reconstruction, signal basis update, signal prediction, registration, and updating the gradient table. Purple boxes showcase the post-processing steps, encompassing T2-weighted image reconstruction, spatial normalization for atlas registration, and streamlined tractography. Refer toSection 2for details on the sampling scheme, dual-echo sequence, motion correction, and dynamic distortion correction.Section 3discusses pre-processing, post-processing, and implementation specifics. Boxes with bold borders refer to our main contributions.

## 2 Material and Methods

HAITCH employs a multi-stage approach to address the challenges associated with fetal dMRI acquisition. Initially, a specialized multi-shell high angular resolution diffusion imaging (HARDI) sampling scheme is designed to increase the overall dataset’s tolerance to motion. Our recommended acquisition involves a modified dual-echo EPI sequence that allows dynamic correction of time-varying geometric distortions. Following acquisition, the data undergo preprocessing, including denoising, Gibbs ringing correction, and Rician bias correction. Geometric distortions are then addressed through non-static field map estimation. Subsequently, B1 bias field correction and fetal brain segmentation are performed. Motion correction is achieved through an iterative refinement process. A flowchart summarizing the various processing stages of HAITCH is shown inFigure 1. This approach progressively improves the data by repeatedly updating slice weights, transform coefficients, and motion parameters, which will be described in detail in the sections that follow. Through these iterative updates, the self-consistency of the data is enhanced, leading to progressively improved motion-corrected images. In this section, we first explain the multi-shell sampling scheme, then we describe the signal representation that makes the foundation of our modeling, sampling, image reconstruction, outlier detection, slice weighting, basis updating, and motion parameters estimation. At the end, we explain the dynamic distortion correction component based on our recommended multi-echo EPI sequence. The details of the experiments conducted, pre-processing, implementation aspects of our methods, and post-processing are explained inSection 3.Section 4will then present the results obtained.

### 2.1 A multi-shell HARDI sampling scheme for the fetal brain

dMRI with multi-shell HARDI provides rich information about the tissue microstructure by varying b-values and directional sampling. Utilizing an extension of the electrostatic repulsion cost function (Caruyer et al., 2013;Dubois et al., 2006), we have customized a sequence with HARDI, interlaced multi-shell, and an incremental sampling scheme for uniform angular coverage. This sampling scheme ensures a balance between the global angular distribution of all diffusion gradient directions and the angular distribution on each shell. The angular density of coverage for each shell increases uniformly as fetal dMRI acquisition proceeds. This optimization of the angular coverage and the temporal ordering aims to mitigate the effects of both sudden and slow fetal motion during acquisition. Acquired slices and volumes with close gradient directions are separated in acquisition time, thereby increasing the motion tolerance of the entire dataset. With this acquisition scheme, any part of the acquired data will contain uniformly distributed orientations, which is not possible with conventional schemes.

Furthermore, to maximize the information content of the data within our multi-shell HARDI acquisition scheme, we determined the per-shell sampling density and the associated b-values based on the detectable number of SH, as detailed below. Previous studies have shown that the angular frequency in neonates is significantly lower compared to adults (Tournier et al., 2013,^2020^).Tournier et al. (2020)demonstrated in a neonatal study that SH terms beyond order 8 are indistinguishable from noise for typical SNR values. SH terms of order 6 were small, suggesting a minimum requirement of 28 directions at high b-values. At lower b-values, only terms of orders 2 or 4 could be detected, implying minimum requirements of 6 and 15 directions, respectively. In practice, acquiring more than the minimum diffusion-weighted directions is highly recommended to account for potential imperfections in the diffusion-encoding gradient directions and ensure sufficient effective SNR for utilizing SH terms with order 6. Additionally,Tournier et al. (2020)determined [0, 400 s/mm^2^, 1800 s/mm^2^] and [0, 400 s/mm^2^, 1000 s/mm^2^, 2600 s/mm^2^] b-value combinations as the optimal for 2-shells and 3-shells sampling schemes in their neonatal imaging study. Their corresponding number of directions expressed as a percentage of the total number of volumes acquired are as follows: [12%, 29%, 59%] and [6%, 19%, 28%, 47%], respectively.

Considering the potential differences and similarities between fetal and neonatal imaging, the dual-echo nature of our sequence, and achievable b-values due to inherent low SNR in fetal dMRI (Karimi et al., 2021), we propose a 2-shell HARDI scheme. This scheme incorporates 11 interleavedb=0images, and diffusion-weighted images atb=400s/mm^2^andb=900s/mm^2^with 28 and 56 directions per shell, respectively.

### 2.2 Signal representation

Diffusion MRI measures the attenuation of MRI signals induced by water diffusion within biological tissues, which is highly dependent on the local microstructure. The accurate modeling and reconstruction of dMRI data require a precise mathematical representation of the diffusion signal,E(q), that can account for the complex interplay of tissue microstructure, diffusion gradient directions, motion artifacts, and geometric distortions. In fetal imaging, the situation is further complicated by low signal-to-noise ratio (SNR) and inherent motion.

#### 2.2.1 Diffusion signal modeling

To effectively capture the orientation-dependent nature of dMRI contrast, we use a set of basis functions, which approximate the observed diffusion signal as a weighted sum of functions with known properties. In this context, we employ the 3D Simple Harmonic Oscillator Reconstruction and Estimation (SHORE) technique (Merlet & Deriche, 2013;Özarslan et al., 2013). Consequently, the dMRI signalE(q), whereqis the wavevector describing the applied diffusion gradient, is expressed as a truncated series expansion over orthonormal basis functions. SHORE allows the diffusion signal to be modeled in both the radial and angular domains, which is particularly important in fetal dMRI, where the complexity of tissue microstructure and motion artifacts demand a flexible and robust representation. The diffusion signalE(q)is modeled as:

$$E\left(\text{q}\right)=\sum_{l=0,\text{even}}^{N_{\text{max}}}\sum_{n=l}^{\left(N_{\text{max}}+l\right)/2}\sum_{m=-l}^{l}{\text{c}}_{nlm}\Phi_{nlm}\left(\text{q}\right)$$ (2)

where the${\text{c}}_{n\ell m}=\langle E,\Phi_{n\ell m}\rangle$are the transform coefficients that capture the contribution of each basis function,$\Phi_{nlm}\left(q,\text{u}\right)=X_{nl}\left(q,\zeta \right)Y_{l}^{m}\left(\text{u}\right)$are the SHORE basis functions and can be decomposed into (i)$X_{nl}\left(q,\zeta \right)$represents the radial part of the signal, modeled by generalized Laguerre polynomials modulated by an exponential decay and scaling factorζ, ensuring orthonormality in the radial domain.nis the radial order. (ii)$Y_{l}^{m}\left(\text{u}\right)$represents the angular part, modeled by SH, which capture the angular dependence of the diffusion signal. Here,lis the degree of the harmonic, andmis its order.$N_{\text{max}}$is the maximal order of the functions in the truncated series.

This transformation offers us two significant advantages to reconstruct and recover motion-corrupted fetal dMRI signal accurately. First, its ability to represent diffusion in tissues with complex fiber structures, such as crossing fibers (Fick et al., 2015). Additionally, the sparsity of the SHORE representation, in which the diffusion signal is captured by only a few coefficients, helps reduce the computational complexity while maintaining accuracy (Merlet & Deriche, 2013). This representation translates the fetal dMRI scan into a compact 4D representation of SHORE coefficients,${\text{c}}_{nlm}$, associated with each voxel in the image grid. With these coefficients and such motion parameters, we can predict the expected fetal dMRI contrasts in the presence of motion using a forward model. This prediction allows us, in turn, to investigate the difference between the estimated and the acquired motion-corrupted fetal dMRI data. Subsequently, we formulate an inverse problem that optimizes the similarity between the predicted and acquired fetal dMRI signal while simultaneously estimating the fetal motion traces.

#### 2.2.2 Forward modeling

Fetal motion during dMRI acquisition introduces significant artifacts and degrades the consistency of the acquired data. Additionally, the diffusion signal is also influenced by geometric distortions arising from inhomogeneities in the main magnetic field$B_{0}$, as well as noise, which can originate from sources such as gradient coils or electronic components. To mitigate these challenges, we utilize a forward model that simulates the combined effects of motion, geometric distortions, and noise on the diffusion signal. By assuming rigid body motion, we model the transformation of both spatial and angular coordinates, and therefore we predict the expected signal under the influence of motion. This forward model establishes a mathematical relationship between the acquired dMRI data, the underlying true diffusion signal, and the motion parameters. It effectively simulates how the diffusion signal would appear after being corrupted by motion.

For a given set of fetal motion parametersμ, the predicted signal$\hat{\text{E}}$could be expressed as:

$$\hat{\text{E}}\left(\mu ,\text{c},\text{q}\right)=\text{c}\cdot \Phi \left(\text{q}\right)\cdot \text{M}\left(\mu \right)\cdot \text{D}\left(\mu \right)\cdot \text{A}$$ (3)

wherecrepresents the transformation coefficients,Aaccounts for noise contributions from various sources,D(μ)models the geometric distortions resulting from$B_{0}$inhomogeneities, which are common in EPI sequences and magnified by fetal motion,M(μ)models the effect of fetal motion on the signal, andΦ(q)encodes the SHORE basis for the givenq-space.

This forward model provides a comprehensive understanding of the complex interplay between motion, geometric distortions, and noise during the acquisition of fetal dMRI data. By simulating how the true diffusion signal is altered, it highlights the importance of accounting for dMRI signal variations in both motion states and diffusion encoding directions. Furthermore, the model underscores how motion exacerbates geometric distortions, demonstrating that static field map estimation is insufficient for accurate distortion correction. As a result, dynamic approaches that consider real-time motion are essential for precise correction. The predicted signal serves as a critical component in the subsequent motion correction and reconstruction process, where it is compared to the acquired data to estimate fetal motion traces and refine transformation coefficients.

### 2.3 Inverse problem and reconstruction

To correct for motion and reconstruct the fetal dMRI signal, we solve an inverse problem. Given the scattered data acquired during the dMRI scan, the objective is to estimate both the optimal transformation coefficients${\text{c}}^{*}$and the unknown fetal motion traces$\mu^{*}$that minimize the discrepancy between the predicted and acquired signals. This involves finding the combination of reconstruction coefficients and motion parameters that best explain the acquired data. This is formulated as a large, sparse least-squares problem with anℓ2regularization:

$$\begin{array}{l} {\text{c}}^{\left(k\right)}=\underset{\text{c}\in {\mathbb{R}}^{{\text{n}}_{\text{c}}}}{\text{arg min}}{\left\|W\left({\hat{\text{E}}}^{\left(k\right)}\left(\mu^{\left(k\right)},\text{q}\right)-\text{E}\right)\right\|}_{\ell 2}\\ \;\;\;\;\;\;\;\;\;+\lambda_{l}{\left\|Lc\right\|}_{\ell 2}+\lambda_{n}{\left\|Nc\right\|}_{\ell 2} \end{array}$$ (4)

where${\hat{\text{E}}}^{\left(k\right)}$is the predicted signal computed from scattered data in the current iterationkusing the coefficients${\text{c}}^{\left(k\right)}$,$W=\text{diag}\left(\cdots ,\;\sqrt{w_{s}},\cdots \right)$,$w_{s}$is a weighting matrix that accounts for slice-wise or voxel-wise outlier rejection. The weighting matrixWassigns higher importance to reliable slices or voxels and reduces the influence of outliers.Nand$L\in {\mathbb{R}}^{n_{q}\times n_{c}}$are two diagonal matrices withdiag(N)=n(n+1)anddiag(L)=l(l+1), respectively. These matrices penalize the high frequencies of the radial and angular components of the SHORE representation.$\lambda_{l}$and$\lambda_{n}$are regularization parameters controlling the strength of these penalties.

Equation 4minimizes a cost function that measures a weighted difference between the predicted signal${\hat{\text{E}}}^{\left(k\right)}$and the acquired signalE. We formulate the solution of this least-squares problem as an iterative process that leverages a data-driven signal representation. We focus on optimizing the target image for maximum similarity to the acquired scattered data, while simultaneously identifying the unknown fetal motion traces that correspond to the fetal brain position. We utilize surrounding directional information to compensate for potential data loss and reconstruct scattered data across spatial and angular domains. The optimization process consists of repeatedly alternating between slice weighting, a reconstruction step optimizing the SHORE coefficientscgiven current motion parametersμ, and a registration step updatingμfor the currentcthroughout each epoch of our framework.

### 2.4 Outlier detection and weighting

Fetal dMRI is inherently susceptible to outliers and imaging irregularities, which can negatively impact image quality and the subsequent processing steps. Outliers are signal intensities that significantly deviate from the expected signal based on other measurements. Fetal motion and maternal breathing during diffusion encoding can induce severe signal dropouts. Moreover, rapid slice acquisition times combined with fetal motion can lead to spin-history artifacts, manifesting as signal hyper-intensities due to overlapping slice excitation. Hence, detecting and appropriately weighting outliers are essential to mitigate the impact of unreliable slices on the reconstruction. To examine the likelihood of a slice$S_{i}$or voxel$V_{i}$with gradientgbeing an outlier, we employ three complementary methods.

#### 2.4.1 Modified Z-score

We employ a modifiedz-score and median absolute deviation (MAD) to detect outliers within each b-value shell. MAD focuses on the median, which is less swayed by outliers, and is a better choice than the standard deviation for outlier detection in datasets that might have outliers. Given a fetal dMRI dataset, let$\text{E}[{\hat{S}}_{i,b,g}]$represent the diffusion signal intensity average for thei-th slice with b-valueband gradient directiong, and let$\text{E}[\tilde{S_{i,b}}]$present the median of those average across the same b-valueb. We compute the modifiedz-score for sliceiwith gradient directiongas follows:

$$Z_{i,g}=\frac{\left|\text{E}[{\hat{S}}_{i,b,g}]-\text{E}[\tilde{S_{i,b}}]\right|}{1.4826\cdot \text{MAD}(\text{E}[{\hat{S}}_{i,b,g}]-\text{E}[\tilde{S_{i,b}}])}$$ (5)

Subsequently, lower$\eta_{l}$and upper$\eta_{u}$thresholds for the modifiedz-scores are applied to assign a weight$w_{s}$to each slice as following:

$$w_{s}=1-\frac{Z_{i,g}-\eta_{l}}{\eta_{u}-\eta_{l}}$$ (6)

These weights are incorporated within the initialization epoch to influence the initial predicted signal${\hat{\text{E}}}^{\left(1\right)}$.

#### 2.4.2 Bayesian Gaussian mixture modeling

The second method utilizes a probabilistic criterion to identify outliers based on the intensity difference between the acquired dMRI data and their corresponding predictions (Christiaens et al., 2021). This approach identifies and excludes outlier slices exhibiting large residuals compared to other slices with similar b-values. We first calculate the root-mean-squared error (RMSE), denoted by∈, between the acquired data and the provided signal predictions. The RMSE values for inlier and outlier slices are expected to follow different probability distributions. Within each shell, a two-component Bayesian Gaussian Mixture Model (GMM) is employed to model the log-transformed RMSE values, thereby delineating inlier and outlier slices. The GMM assigns a probability, considered as$w_{s}$, to each slice belonging to the inlier component.

#### 2.4.3 Voxel-wise weighting

The previous two methods focused on slice-level weighting to address signal dropout caused by bulk motion during acquisition. However, local variations within slices can arise due to factors like spin history and physiological motion. To down-weight these localized outliers, we employ a voxel-wise weighting applied in the final processing epoch. This method builds upon the voxel-wise standardized residuals with the modified z-score which are then converted to weights for each voxel as follows:

$$w_{v}=\frac{1}{{\left(z_{v}^{2}+1\right)}^{2}}\;\text{where}\;z_{v}=\frac{E_{v}-{\hat{E}}_{v}}{1.4826\cdot \text{MAD}\left(E_{v}-{\hat{E}}_{v}\right)}$$ (7)

Here,$E_{v}$and${\hat{E}}_{v}$represent the acquired and predicted signal intensity for a particular voxel, respectively.

### 2.5 Registration

In this step, we seek to compute and update the motion parameters by optimizing the spatial alignment between the predicted signal and the acquired scattered data. We achieve this by performing rigid image registration directly between the predicted and the acquired dMRI contrasts at each time point. This approach offers several advantages over conventional registration methods that often use the b0 image (unweighted image) as the reference for aligning the acquired dMRI data. Here, the predicted dMRI contrasts at each time point serve as the reference for registration with the acquired dMRI data. This way of registration is more accurate than the conventional registration to b0 images, as the fixed (predicted) and moving (acquired) images have the same diffusion sensitization.

### 2.6 Dynamic distortion correction

Fetal dMRI often relies on EPI for its efficient data acquisition. However, EPI inherits a limitation: the presence of local geometric distortions. These distortions are a result of the inhomogeneities of the main magnetic field B0, which are caused by the differences in magnetic susceptibility of tissue and air/gas in air/gas-tissue interfaces. A common approach to correct geometric distortions in adult dMRI involves acquiring scans with a single phase encoding direction and adding a*b*= 0 image with reversed phase encoding. A field map, assumed to be static, is then generated from the data and used for correcting distortions (Andersson et al., 2003). While effective for subjects with minimal motion, this method is inadequate for fetal imaging because fetal motion affects field inhomogeneities. The nearly-continuous fetal movements as well as gas movement in the maternal intestine, and maternal respiratory motion, can cause significant changes in B0 inhomogeneity. These changes would render static field map correction techniques inaccurate in fetal dMRI (Afacan et al., 2019,^2020^;Hutter et al., 2018;Kuklisova-Murgasova et al., 2017).

**Fig. 2.**
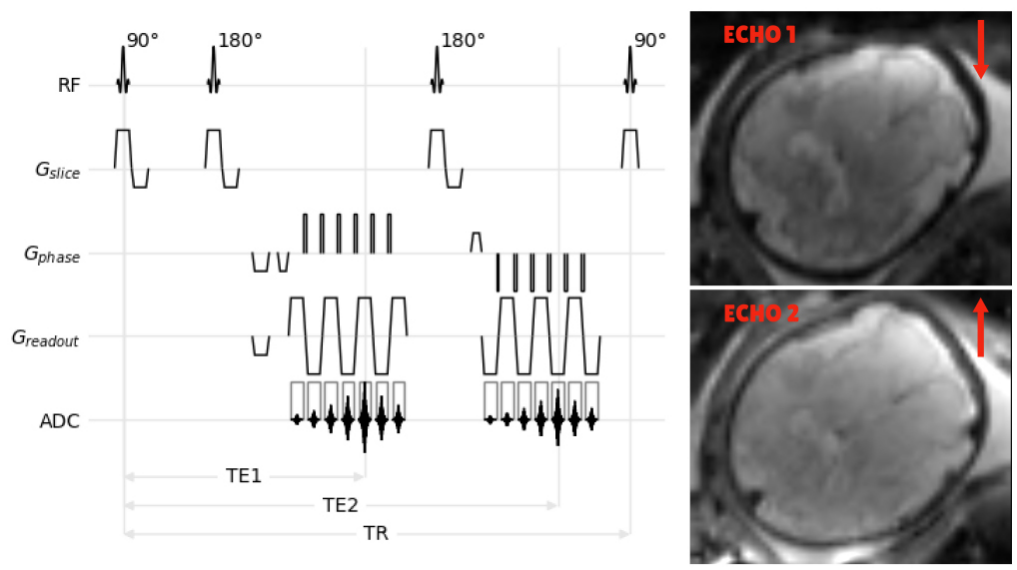
(Right): Schematic diagram of the modified spin-echo EPI sequence. This sequence acquires two echoes (TE1 and TE2) with reversed phase encoding directions. Despite the extended readout, the dual-echo sequence maintains signal-to-noise ratio (SNR) efficiency comparable to a conventional EPI scan. Temporal proximity between the echoes (<50 ms) ensures minimal motion artifacts between acquisitions. (Left): Illustration of the opposing susceptibility-induced distortions in the two echoes for an example. The first echo (TE1) exhibits stretching (arrow), while the second echo (TE2) shows signal pile-up in the corresponding location. This key feature allows for dynamic field map estimation and correction of geometric distortions even in the presence of fetal motion. Details of the implementation are discussed inSection 3.

To address this challenge, we propose using a modified spin-echo EPI sequence incorporating a second readout (echo). This second echo generates data that have the same resolution, field of view, and bandwidth of the first echo data but with the order ofk-space traversal reversed in the phase encoding direction.Figure 2illustrates the pulse sequence diagram. A 180° refocusing pulse and spoiler gradients were employed to generate the second spin-echo. The frequency encoding direction is reversed between the two echoes to ensure consistent temporal spacing betweenk-space points. Notably, the dual-echo sequence maintains the SNR efficiency comparable to the conventional EPI scan despite the extended readout train. The temporal proximity between the two echoes (<50 ms) ensures closely matched and nominally identical motion states for the two acquired slices. Importantly, the first and second echo-time data exhibit geometric distortions in opposite directions at each acquisition time point.Figure 2further illustrates this concept, where susceptibility-induced stretching in the first echo corresponds to signal pile-up in the second echo. This enables the application of the blip-reversed approach in dynamic environments such as fetal and neonatal imaging. This key advantage allows us to estimate a dynamic field map, thereby correcting for geometric distortions in the presence of motion. Implementation details are discussed inSection 3.

## 3 Implementation and Experiments

### 3.1 Fetal datasets

To examine the efficacy of our proposed framework, we conducted 36 dMRI studies on 14 pregnant volunteers, scanned at a gestational age between 24 and 36 weeks. The studies were approved by the institutional review board, and written informed consent was obtained from all participants. Each scan session involved repeated single-shot fast spin echo T2w acquisitions, which were used to reconstruct a reference anatomical volume with an isotropic resolution of 0.8 mm^3^using SVR (Ebner et al., 2020;Uus et al., 2020). These T2w volumes were essential for alignment.

Each scan session also includes a dMRI scan, which was acquired in three different configurations to test the robustness of our HAITCH framework

- *(i) Dual-Echo Data*: Twenty-four dMRI scans were acquired from 13 subjects using a dual-echo EPI sequence. The first echo time (TE1) was 50 ms, while the second echo time (TE2) varied between 85 ms and 90 ms. These scans included various single-shell and double-shell diffusion-weighting values (b-values) of 250, 400, 500, 600, 750, 800, 900, and 1000 s/mm^2^, across six different direction sets ranging from 13 to 93 gradient directions (13, 15, 20, 22, 26, 27, 44, 80, and 93 directions).
- *(ii) Multi-Shell HARDI Data*: Nine multi-shell HARDI scans were acquired from five volunteers. These scans were acquired following the optimized multi-shell HARDI sampling scheme described inSection 2.1. This scheme included b-values of 400 and 900 s/mm^2^, with 28 directions for the lower shell and 56 directions for the higher shell. The scheme also includes 11 b = 0 volumes.
- *(iii) Dual-Echo Multi-Shell HARDI Data*: An additional three scans were acquired from two volunteers using a combination of the dual-echo EPI sequence and the multi-shell HARDI sampling scheme, as outlined inSection 2.1: 11 volumes with b = 0, 28 volumes with b = 400 s/mm^2^, and 56 volumes with b = 900 s/mm^2^. Acquisition time is approximately 9 min.

All scans were conducted on a 3T Siemens Prisma scanner equipped with a 30-element body coil. Parallel imaging with a GRAPPA acceleration factor of 2 was used to reduce acquisition time. The minimum repetition times (TR) varied between 2.5 and 7 s. Scans were performed with isotropic resolutions of 2.0 mm, 2.3 mm, or 2.5 mm, and a field of view of 256 mm or 300 mm, depending on the gestational age and size of the fetus as well as the maternal anatomy. Each scan session consisted of between 30 and 40 slices to ensure full coverage of the fetal brain. In the dual-echo EPI sequence, the two readouts were acquired with opposite phase-encoding directions; for example, one along the anterior-to-posterior (A → P) axis and the other along the posterior-to-anterior (P → A) axis. This approach helps to mitigate susceptibility-induced distortions by providing data in both directions, which can later be used for distortion correction during post-processing.

### 3.2 Pre-processing

dMRI data is susceptible to various artifacts arising from the limitations of the MRI hardware and techniques. To address these artifacts and ensure reliable estimation of diffusion parameters, we have established the following steps, as represented by green boxes inFigure 1:

#### 3.2.1 Denoising

The data are initially denoised using the generalized singular value shrinkage (GSVS) method for noise estimation and reduction, as detailed inCordero-Grande et al. (2019). This method extends the random matrix theory-based approach of the Marchenko-Pastur Principal Component Analysis (MPPCA), introduced inVeraart, Fieremans, et al. (2016)andVeraart, Novikov, et al. (2016). GSVS moves beyond the limitations of MPPCA, including the homoscedastic and uncorrelated noise assumptions and the hard separability of signal and noise. Instead, it utilizes the findings ofBenaych-Georges and Nadakuditi (2012)for optimal singular value shrinkage under a generalized Marchenko-Pastur law. This makes GSVS particularly suitable for motion-degraded and low SNR data, including fetal dMRI data with high motion artifacts. GSVS effectively removes only thermal noise without compromising anatomical features in approximately 2 min.

#### 3.2.2 Gibbs ringing artifact correction

High-contrast boundaries, such as the edges between cerebrospinal fluid and gray matter or white matter in the fetal brain, may produce image artifacts known as Gibbs ringing. These artifacts arise from inadequate sampling of high frequencies, and may significantly impact the diffusion signal. To remove these artifacts, we employ a method that modulates the truncation ink-space as a convolution with a sinc-function in image space and interpolates the image through local subvoxel shifts (Kellner et al., 2016).

#### 3.2.3 Rician bias correction

dMRI images exhibit Rician or non-centralχnoise, especially in low-SNR settings like multi-shell HARDI fetal dMRI. Unlike Gaussian noise, Rician noise is non-additive and intensity-dependent, leading to Rician bias and reduced image contrast. To address this, we adapt the methodology introduced inAdes-Aron et al. (2018)with GSVS replacing MPPCA for Rician bias correction. GSVS denoising provides an unbiased estimate of the noise standard deviationσat each voxel based on data acquired with low b-values (Cordero-Grande et al., 2019;Veraart, Fieremans, et al., 2016). We then estimate the true signal intensity$E_{c}$through the following equation:

$$E_{c}^{2}={\langle M\rangle }^{2}-\left(\xi \left(\theta \right)-2\right)\sigma^{2}$$ (8)

Here,〈M〉represents the expected value of the measured magnitude signal intensity, andξ(θ)is a correction factor defined by$\theta =\frac{\eta }{\sigma }$, which is equivalent to the SNR (Koay & Basser, 2006). ForSNRexceeding 2 dB,ξ(θ)approaches 1, allowing for accurate signal intensity estimation.

### 3.3 Dynamic distortion correction

Both the first echo time dMRI(TE1) scan and the second echo time dMRI(TE2) scan were used to correct the distortion following the reversed gradient polarity technique. To ascertain the sequence’s efficiency and effectiveness, distortion correction was approached via two dynamic and one static correction methods: (i) Slice-wise correction: field map is estimated for each slice using the reversed echo time slice at every time point for low and high b-values images using the method introduced inVoss et al. (2006). (ii) Volume-wise correction: field map is estimated for each volume using the reversed echo time volume at every time point for both low and high b-values images using FSL TOPUP (Andersson et al., 2003). (iii) Static map correction: the classic way of distortion correction with FSL TOPUP, employing the first b = 0 image of TE1 and the last b = 0 image of TE2 to calculate the static field map, which is then applied to the entire TE1 dataset. These three correction methods were applied reciprocally between dMRI(TE1) and dMRI(TE2), with outcomes evaluated via the Structural Similarity Index Measure (SSIM) and Peak signal-to-noise ratio (PSNR) metrics. The method with the highest SSIM and PSNR was chosen for subsequent processing.

### 3.4 B1 bias field correction

Fetal dMRI data acquired with high-density coils suffer from signal variations due to B1 field inhomogeneity. This is particularly problematic in multi-shell acquisitions where complex anatomy and varying diffusion weightings magnify these effects. To address this, we employ the N4ITK algorithm (Tustison et al., 2010) to estimate the corrected signal$E_{c}\left(\text{q}\right)$as follows:

$$E_{c}\left(\text{q}\right)=E_{DC}\left(\text{q}\right)\cdot \frac{{\hat{E}}_{DC\left(N4\right)}\left(\text{b}=\text{0}\right)}{\beta \cdot {\hat{E}}_{DC}\left(\text{b}=\text{0}\right)}$$ (9)

where$E_{DC}\left(\text{q}\right)$is the distortion-corrected dMRI signal,βis the bias field estimated using the N4ITK from the masked average of all b = 0 images, and${\hat{E}}_{DC}\left(\text{b}=\text{0}\right)$and${\hat{E}}_{DC\left(N4\right)}\left(\text{b}=\text{0}\right)$are the estimated average intensity of b = 0 images before and after applying the N4ITK bias correction respectively.

### 3.5 Fetal brain segmentation

Fetal dMRI scans encompass not only the fetal brain but also surrounding maternal structures. Efficient background removal is essential for enhancing the reliability of subsequent processing steps. However, fetal brain extraction is challenging due to factors like variable fetal head position, movement during scans, and the diverse appearance of the developing fetal brain with adjacent anatomy. To address these complexities, we leverage a deep learning-based method built upon a fully convolutional neural network architecture similar to U-Net++ (Karimi et al., 2020). The model was trained following the established strategy recommended by nnU-Net (Isensee et al., 2021) and has been extensively validated on manually labeled data. This approach effectively removes background structures and performs image cropping to reduce processing time.

### 3.6 Motion correction and reconstruction

The HAITCH framework employs an iterative motion correction and reconstruction process that spans five epochs. Each epoch alternates between outlier reweighting, SHORE coefficients computation, basis updating, signal prediction, registration, and gradient table rotation steps.

The process begins with slice weighting using the modified*Z*-score method, which does not require a pre-existing predicted signal. The reconstruction step then estimates the coefficientscby minimizing the cost function ofEquation 4. The computation of these coefficients involves the use of the SHORE basis of order 6 defined byMerlet and Deriche (2013). It is important to note that this basis differs from the one available in the Dipy library (Garyfallidis et al., 2014), resulting in a greater number of basis functions (72 in Merlet & Deriche vs. 50 in Dipy for radial order 6). The scale parameterζinEquation 2is fixed to a value of 700, and the regularization parameters$\lambda_{l}$and$\lambda_{n}$inEquation 4are set to$10^{-8}$as recommended byMerlet and Deriche (2013). Using these coefficients and the pre-defined sampling scheme, we predict the dMRI signal which, in turn, is used as a reference for the registration step. Rigid registration is performed using ANTs (Avants et al., 2009) to compute motion parametersμfrom the acquired data. This involves volume-to-volume registration, where the fixed and moving images have the same diffusion contrast. A global correlation metric is utilized to guide the registration process. Based on the estimated motion parameters, the gradient directions are subsequently rotated individually.

A new epoch commences with slice weighting using the GMM method, which compares the registered data with the predicted signal. The dMRI signal is defined within aq-space, and to accurately compact this signal, the correct basis must first be computed based on the currentq-space configuration. As the gradient directions, that is,q-space, were rotated according to the estimated motion traces, we update the basis functions by computing$\Phi_{nlm}\left({\text{q}}^{\left(k\right)}\right)$. The reconstruction step then estimates new coefficients in$\Phi_{nlm}\left({\text{q}}^{\left(k\right)}\right)$, and predicts a new dMRI signal in the initialq-space. This newly predicted signal is subsequently used in the next registration phase. As outlined inEquation 2, updating the basis in this iterative manner ensures that we obtain coefficients corresponding to the correct basis functions. In addition, the dMRI signal should be predicted in the originalq-space to be aligned with the acquired dMRI data for proper registration.

Therefore, motion parametersμare updated and refined over four epochs. The final epoch utilizes these refined parameters to obtain final registered data, followed by a voxel-wise outlier detection. Subsequently, SHORE coefficients are computed in the updated basis$\Phi_{nlm}\left({\text{q}}^{\left(5\right)}\right)$, and the process concludes by predicting the final dMRI signal in the original sampling scheme.

### 3.7 Spatial normalization to atlas space

Following the motion correction and reconstruction steps, the HAITCH framework performs spatial normalization to a common reference space. This process involves two stages of registration. First, the motion-corrected and reconstructed dMRI data of the subject are registered to a reconstructed T2w image of the same subject in the world coordinates. The T2w image is obtained using a super-resolution SVR algorithm, either SVRTK (Uus et al., 2020) or NiftyMIC (Ebner et al., 2020). Second, the T2w image is registered to the publicly available CRL fetal brain atlas (Gholipour et al., 2017). This atlas provides a standardized anatomical reference for the fetal brain.

Finally, the two transformation matrices obtained from these separate registrations are combined into a single transformation, which is then applied to the dMRI data to map it into the atlas space while upsampling it to an isotropic voxel resolution of 1.25 mm. These upsampled dMRI data are used in subsequent post-processing steps, including fiber tractography.

### 3.8 Diffusion model estimation and tractography

White matter tractography was conducted utilizing the MRtrix3 toolbox (Tournier et al., 2019). Initially, white matter, gray matter, and cerebrospinal fluid response functions were estimated using the unsupervised method described inDhollander et al. (2019). Subsequently, multi-shell multi-tissue constrained spherical deconvolution was performed to estimate fiber orientation distribution (FOD) maps (Jeurissen et al., 2014). Streamline tractography was then conducted using the iFOD2 algorithm, a probabilistic tracking method that utilizes second-order integration of the FOD maps (Tournier et al., 2010). Tracking parameters were carefully chosen to optimize the tractography and generate accurate white matter pathways from the fetal FOD maps. These included an angle threshold of 45°, a cutoff value of 0.01, a specified number of streamlines (100,000), and an FOD power raised to 6 for enhanced specificity. Additionally, minimum and maximum track lengths were set to 10 and 120 mm, respectively, to target relevant white matter tracts.

### 3.9 HAITCH: Open-source framework

As mentioned earlier, HAITCH framework is publicly available as the first module of our FEDI toolbox, accessible athttps://fedi.readthedocs.ioandhttps://github.com/FEDIToolbox. This toolbox includes both the HAITCH implementation and our optimized sampling scheme for fetal dMRI, making these resources readily available to the research community. HAITCH demonstrates its versatility by effectively handling various fetal dMRI data types, including single-shell, multi-shell, or single-echo acquisitions. It further empowers users with a range of options for customization. These include additional outlier detection methods and alternative registration methods and metrics, allowing researchers to tailor the framework to their specific needs. For dynamic distortion correction, besides the methods mentioned previously, users can easily switch to alternative approaches like Block-Matching (Hedouin et al., 2017) or EPIC (Holland et al., 2010) methods.

To take advantage of the full capacity of HAITCH, we recommend using a blip-reversed dual-echo EPI sequence. We have proactively shared our in-house built dual-echo spin echo EPI sequence (developed by co-authors OA and MO) on the Siemens R2C platform. This ensures that researchers will have all the necessary tools readily available for successful HAITCH implementation and application in their fetal brain imaging studies.

## 4 Results

### 4.1 Distortion correction: Dynamic field map estimation

**Fig. 3.**
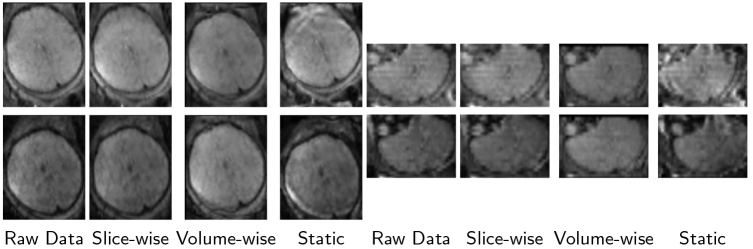
Comparison of Fetal dMRI Distortion Correction Techniques. This figure illustrates the impact of different distortion correction techniques on fetal dMRI data. The top row displays dMRI (TE1) and the bottom row shows dMRI (TE2) images. Left and right panels represent axial and coronal views, respectively. Each column showcases the results for a different dataset or correction technique: Raw Data: Uncorrected image exhibiting distortions due to susceptibility variations. Slice-wise Correction: Distortion correction is applied to individual slices independently. Volume-wise Correction: Distortion correction is simultaneously applied to the entire volume (HAITCH approach). Static Correction: Correction based on a static field map, potentially inaccurate due to fetal motion.

**Fig. 4.**
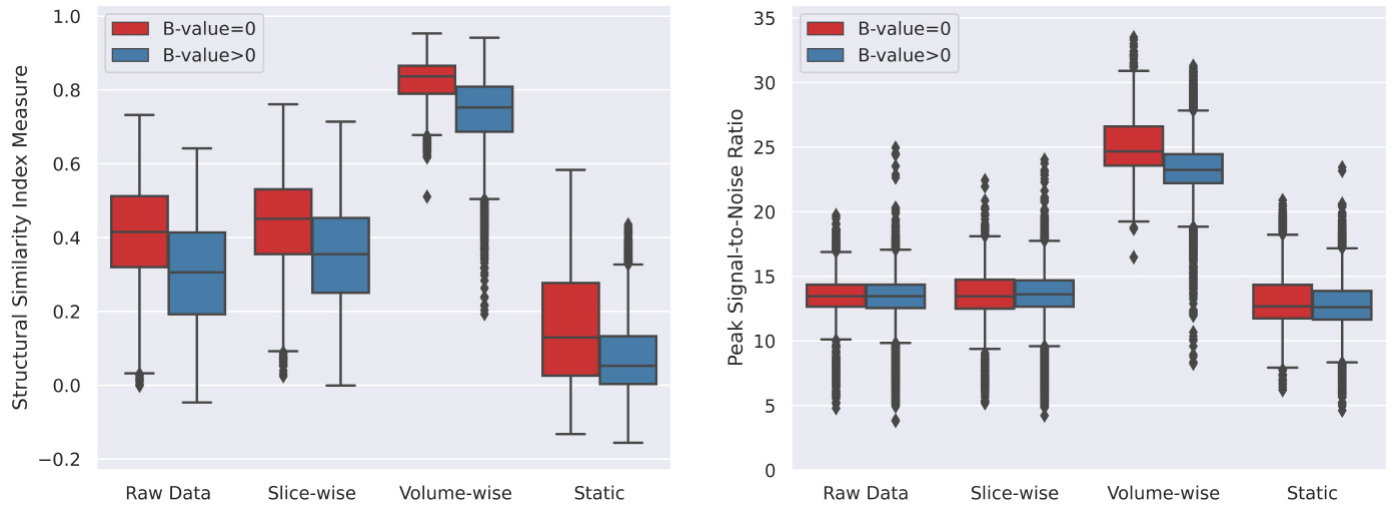
Quantitative Evaluation of Distortion Correction Techniques using SSIM and PSNR. Boxplots summarize the distribution of SSIM (left) and PSNR (right) values across 27 subjects. Results are presented for raw data and data processed with: Slice-wise correction, Volume-wise correction (HAITCH approach), and Static field map correction obtained from the three echo reversal correction methods. Red boxes represent b = 0 images, while blue boxes display diffusion-weighted images (b>0). Higher SSIM and PSNR values indicate better image quality and reduced distortion. As evident from the plots, the volume-wise dynamic correction method achieved the best results across both metrics and image types.

Field map estimation utilizing both dynamic and static echo reversal correction approaches was conducted on 27 dMRI scans. The effectiveness of each correction method was evaluated visually and quantitatively.Figure 3presents an illustrative example of distortion correction achieved for each method. Overall, visual inspection shows that volume-wise dynamic correction is superior in recovering anatomical brain shape compared to the raw data and other correction techniques. Corrected dMRI (TE1) and dMRI (TE2) appear to have greater visual similarity than those processed with other methods. To quantify these observations,Figure 4illustrates the SSIM and PSNR statistics for both b = 0 andb>0images across all slices for all scans. The analysis reveals that both dynamic field map estimation methods have significantly enhanced the SSIM and PSNR values compared to the raw data (uncorrected). The volume-wise approach yielded the highest SSIM and PSNR, indicating superior performance in correcting geometric distortions. Conversely, static field map-based correction resulted in lower SSIM and approximately similar PSNR compared to raw data. This highlights its ineffectiveness in addressing motion-induced field inhomogeneities.

### 4.2 Evaluation of the motion correction

**Fig. 5.**
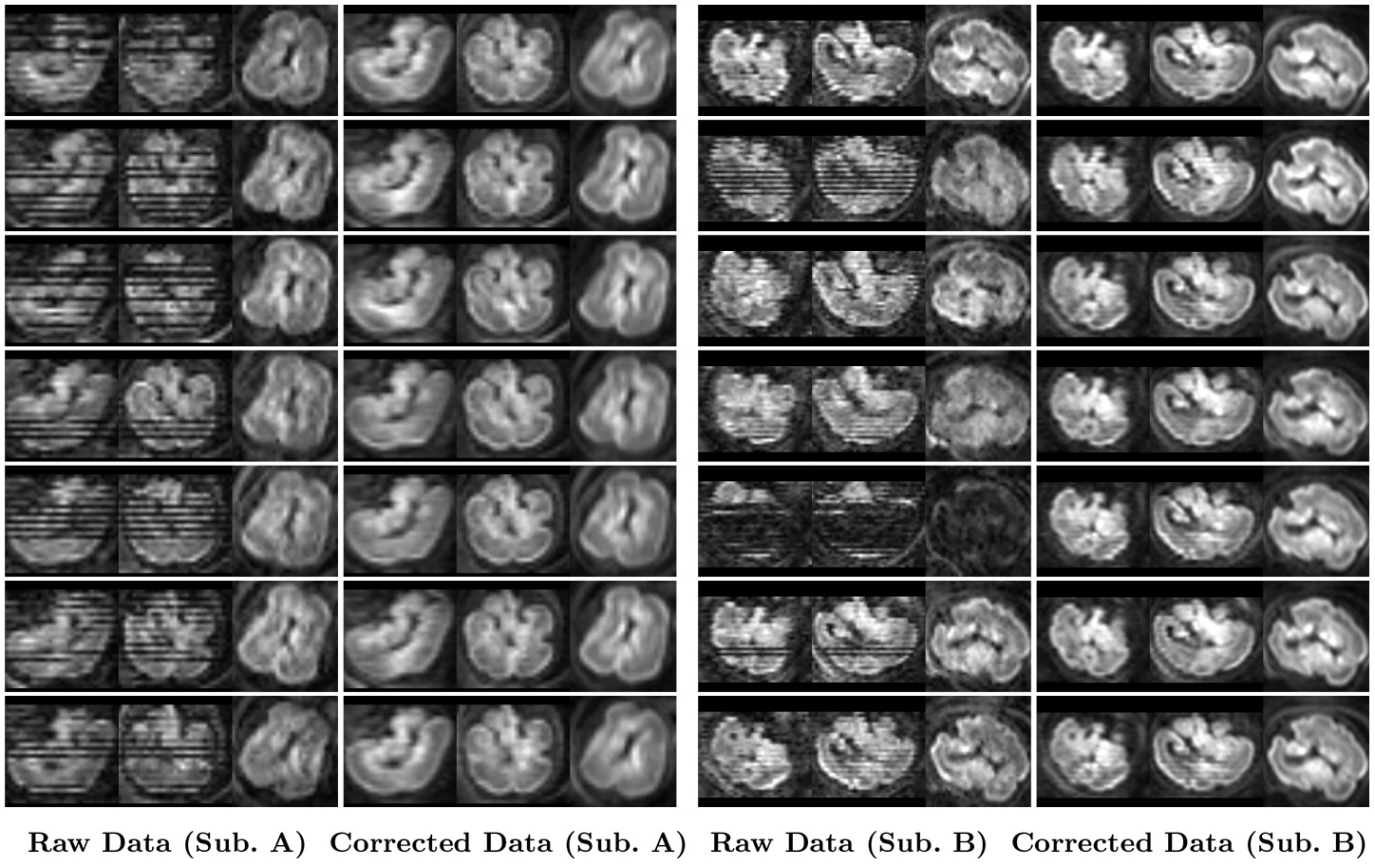
Example fetal dMRI scans before and after motion correction. The left two columns display axial, coronal, and sagittal views of the raw data (pre-processed up to B1 bias field correction step) (Subject A) and corresponding motion-corrected data, respectively. Each row represents a specific volume (index: 5, 8, 15, 18, 27, 30, 31 from top to bottom). The right two columns show corrected data for Subject B. Each row represents a specific volume (index: 7, 37, 38, 50, 58, 60, 87 from top to bottom).

**Fig. 6.**
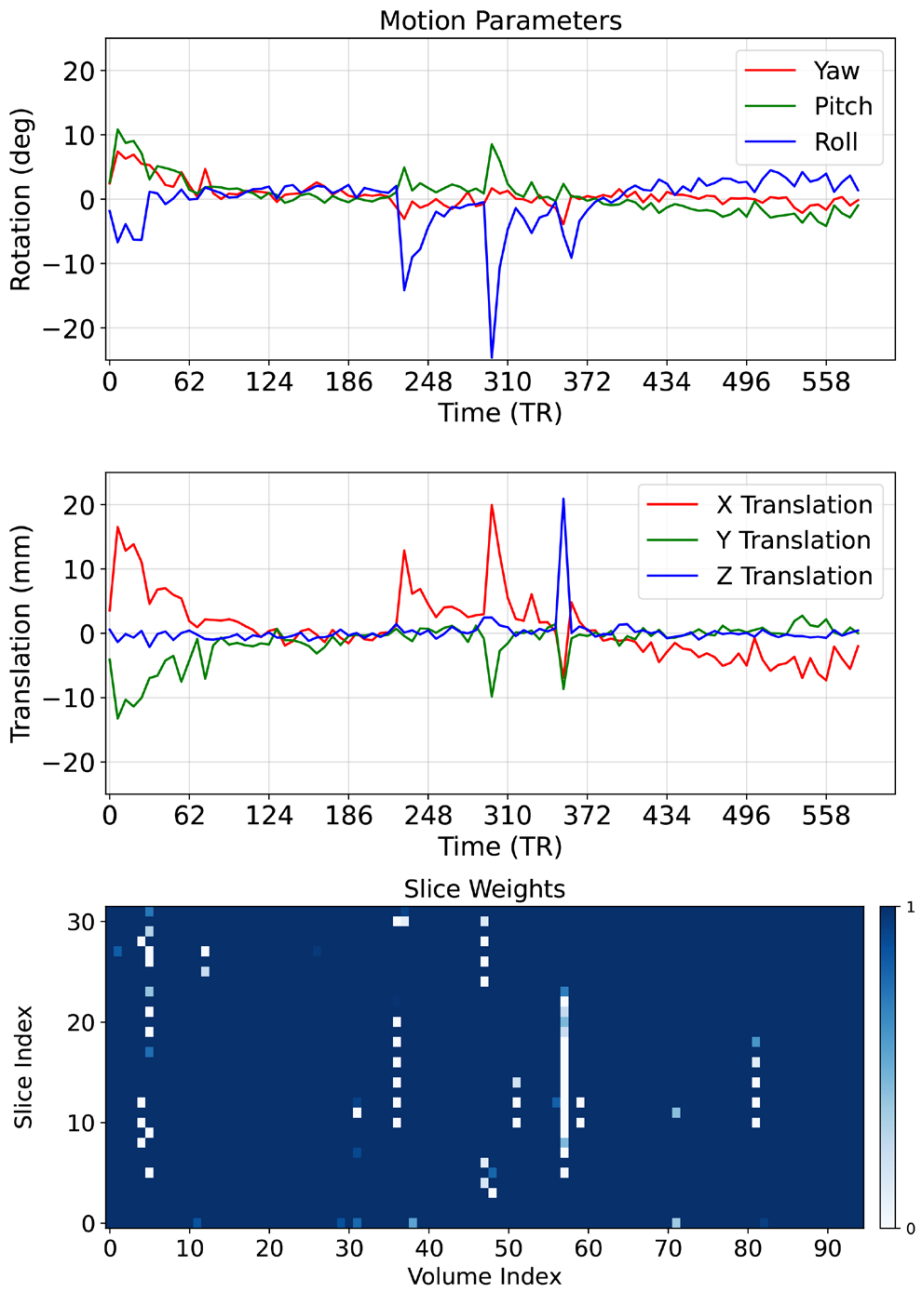
Estimated motion parameters over time and slice weights for Subject B: The top two panels show the estimated motion parameters: translation (mm) and rotation (Euler angles). The bottom panel displays the slice weights calculated using the modified*Z*-score method. Peaks in motion parameters coincide with low slice weights (outliers).

Figure 5showcases the effectiveness of the motion correction stage of our framework by comparing motion-corrupted fetal dMRI data (first and third columns) with their corresponding motion-corrected reconstructions (second and fourth columns) for two subjects*A*and*B*. The raw data exhibit significant motion artifacts, including signal dropouts and inconsistencies in head orientation across multiple volumes. In contrast, the motion-corrected images inFigure 5(second and fourth columns) show dramatic improvements. Signal intensity is successfully recovered, revealing clear visualization of the fetal brain anatomy across all three orthogonal views (axial, coronal, and sagittal). This visual comparison emphasizes HAITCH’s capability to effectively mitigate motion artifacts, and leads to improved data quality suitable for further analysis. To gain deeper insights into the motion correction process,Figure 6depicts the estimated motion parametersμfor subject*B*. The observed translations range from -12 mm to 20 mm, and rotations range from -24 to 10 degrees. The motion profile reveals a slowly varying baseline started by one extended period and punctuated by three shorter bursts of rapid motion. These transient motion events correspond to regions with low slice weights in the lower panel ofFigure 6, as identified by Z-score. This alignment highlights the efficacy of the HAITCH motion correction stage, as it successfully accounts for inter-volume head motion inconsistencies, corrects for intra-volume motion artifacts, and ultimately recovers signal intensity and improves slice uniformity.

**Fig. 7.**
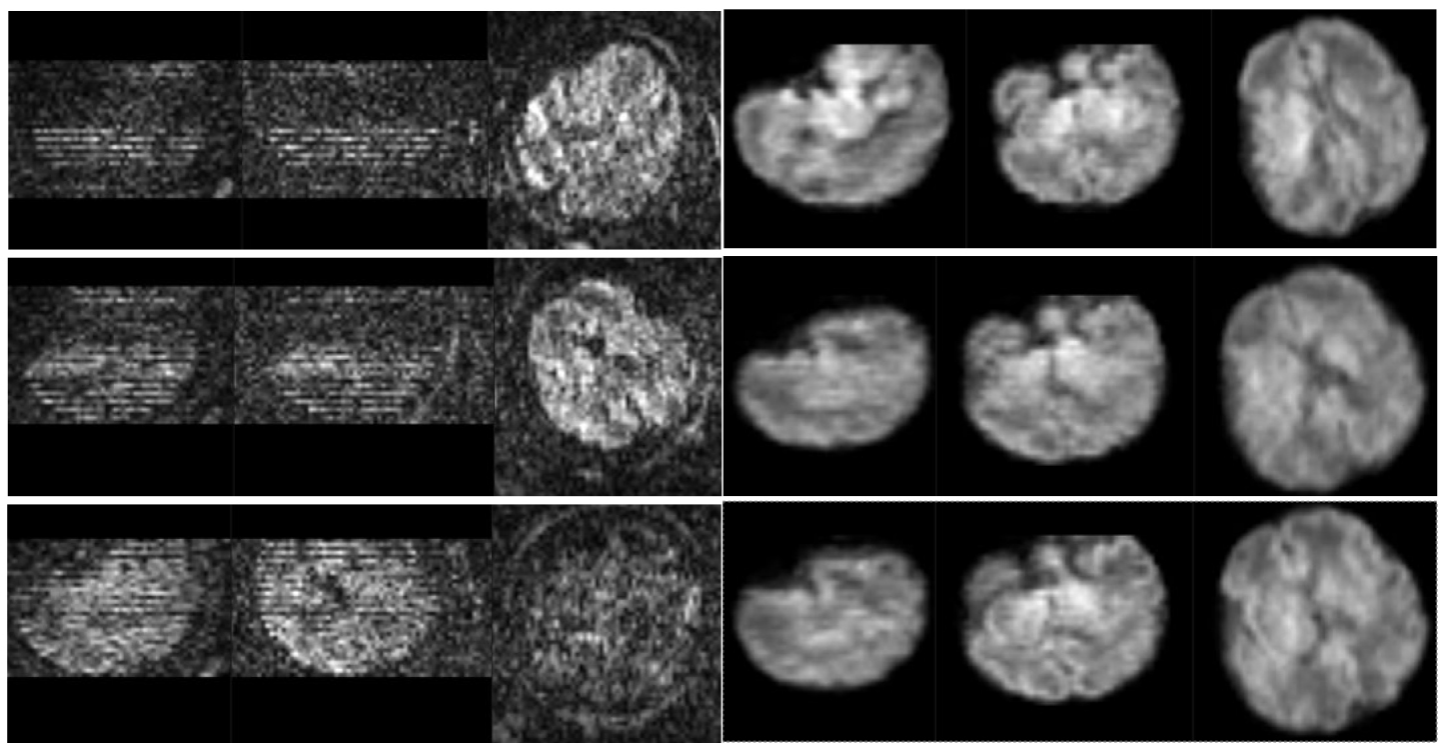
Fetal dMRI with Motion Correction in Challenging Scenario (Subject C). The left column shows raw data from three volumes with close temporal proximity (index: 33, 46, 49 from top to bottom) with significant outliers, signal dropouts, and various brain orientations. The right column displays the corresponding masked reconstructed volumes with improved anatomical details.

Figure 7showcases HAITCH performance even in challenging scenarios, such as low SNR data exemplified by subject C. The figure displays three volumes with close temporal proximity and various brain orientations. Despite this, our framework successfully recovers anatomical details. The well-defined masked brain in the right panel ofFigure 7also demonstrates the robustness of our deep learning model in fetal brain segmentation. Note that segmentation can be performed before or after motion correction depending on the registration stage which can be sometimes better when keeping the background.

**Fig. 8.**
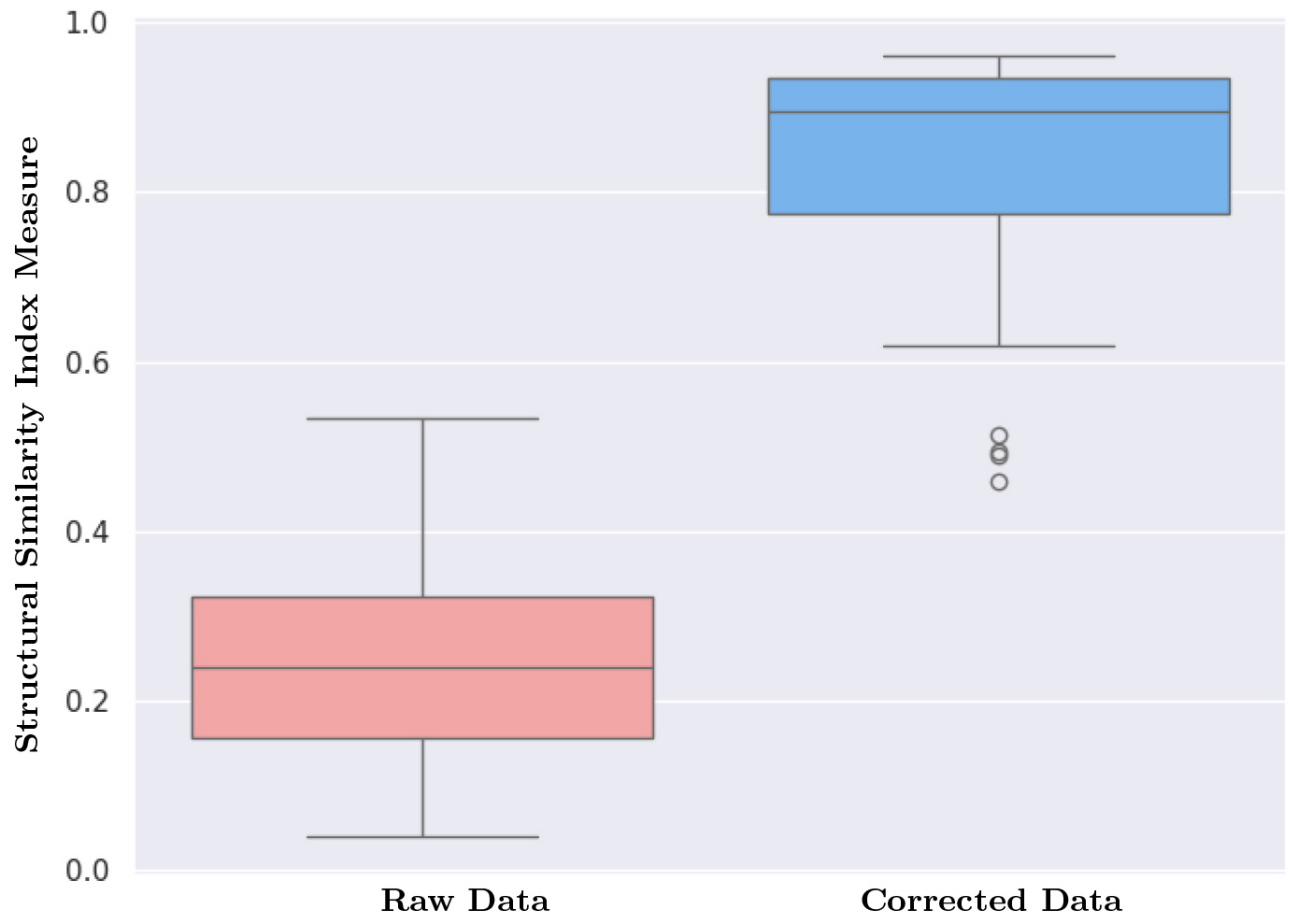
Distribution of SSIM Values for Raw and Motion-Corrected dMRI. Boxplots compare the SSIM distribution across slices of the first and last volume (b-value = 900 s/m^2^) for eight subjects. Raw data (red) exhibit lower SSIM compared to motion-corrected data (blue), indicating improved data integrity after motion correction.

Furthermore, we employed quantitative analysis to complement the visual assessment.Figure 8presents the distribution of SSIM values for raw and motion-corrected data across slices between the first and last volumes (b-value = 900 s/mm²) for eight subjects. The initial and final volumes represent a significant temporal gap during the scan, making them particularly susceptible to intra-scan motion. Our analysis revealed a significant increase in SSIM values after motion correction, which indicates enhanced data integrity and reduced artifacts. This quantitative finding reinforces the visual observations of improved anatomical detail and strengthens the overall validation of the HAITCH framework.

### 4.3 Microstructure mapping

**Fig. 9.**
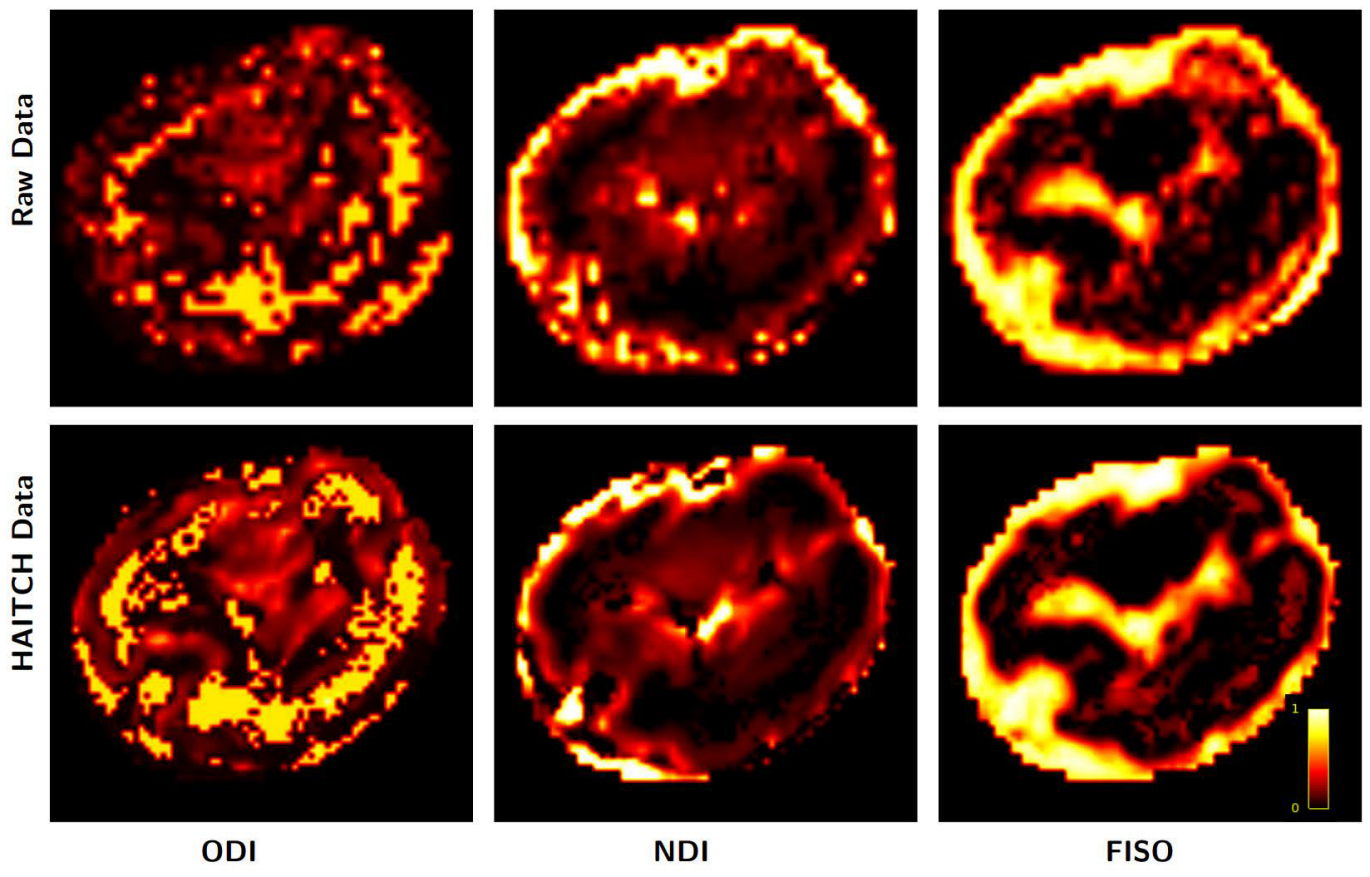
Comparison of NODDI parameter maps before and after applying the HAITCH correction. The top row shows the raw NODDI maps derived from uncorrected diffusion MRI data, while the bottom row shows the maps after motion and distortion correction using HAITCH. From left to right: Orientation Dispersion Index (ODI), Neurite Density Index (NDI, also referred to intracellular volume fraction - ICVF), and Free Water Volume Fraction (FISO). Detailed discussion of these results is provided inSection 5.

Neurite Orientation Dispersion and Density Imaging (NODDI;Zhang et al., 2012) is a widely used dMRI model that characterizes microstructural properties within each voxel by estimating three key scalar parameters: (i) Neurite Density Index (NDI) quantifies the proportion of tissue volume occupied by neurites (axons and dendrites) within a voxel. (ii) Orientation Dispersion Index (ODI) describes the angular variability of neurite orientations. It ranges from 0 (perfectly aligned, straight fibers) to 1 (completely isotropic, randomly oriented fibers). (iii) Free Water Volume Fraction (FISO) represents the fraction of the voxel volume that is occupied by extracellular free water, which is not restricted by tissue microstructure.Figure 9illustrates NODDI-derived microstructural maps for the uncorrected (raw) data and the data processed using the HAITCH framework for subject*B*(gestational age is 28.43 weeks). Motion level for this subject is considered medium, comparing for example to what we have inFigure 7. The HAITCH-corrected data provide improved estimates of NDI, ODI, and FISO by significantly reducing artifacts and noise, which can otherwise bias the parameter estimates. A more detailed discussion of these results is provided inSection 5.

### 4.4 Anatomical accuracy

**Fig. 10.**
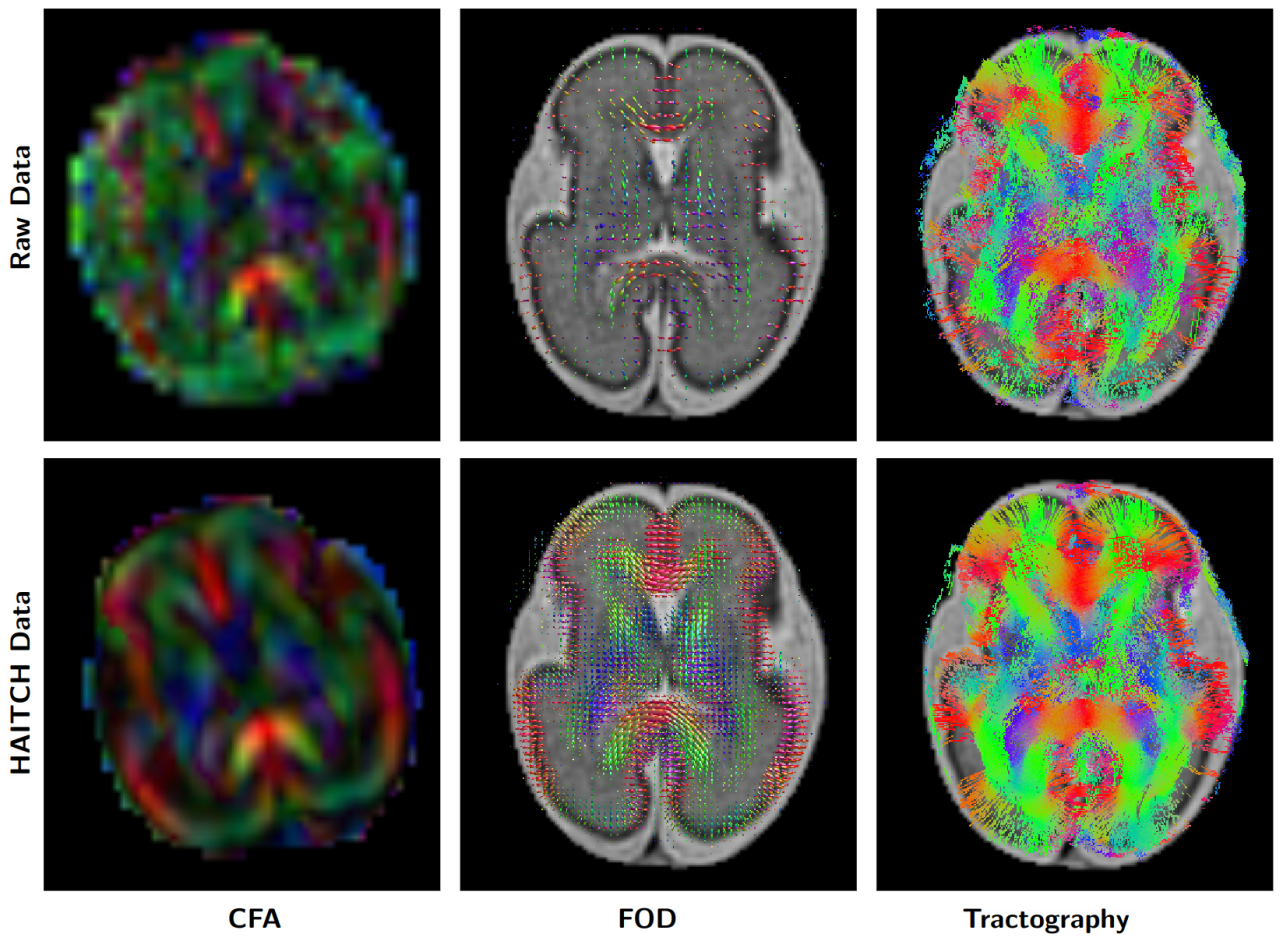
Comparison of Anatomical Accuracy Before and After HAITCH Correction. The top row shows the raw data, while the bottom row shows the HAITCH-corrected data. From left to right: Colored Fractional Anisotropy (CFA) maps, Fiber Orientation Distributions (FOD), and tractography streamlines. The FODs are overlaid on a T2-weighted image in the middle column, and the tractography streamlines are shown in the right column. The images depict the brain of a 30-week fetus, with improved alignment of the white matter pathways after motion correction, demonstrating the efficacy of HAITCH in reducing artifacts and enhancing anatomical accuracy.

Assessing the alignment of derived white matter pathways with structural scans provides valuable insights into the effectiveness of the HAITCH framework. Specifically, comparing Fiber Orientation Distributions (FOD) and tractography streamlines with structural T2w images offers a qualitative evaluation of anatomical accuracy.Figure 10illustrates how well the FODs align with the developing white matter structures, with tractography streamlines overlaid on the T2w image. This improved alignment suggests that HAITCH successfully provides reliable dMRI data suitable for studying fetal brain development, even in the presence of significant motion artifacts. The figure also includes Colored Fractional Anisotropy (CFA) maps derived from Diffusion Tensor Imaging (DTI), which further highlight the enhanced directional coherence of the white matter tracts. Moreover,Figure 10demonstrates the improvement in connectivity, sharpness, and overall structure between the raw data and the HAITCH-corrected data, across CFA, FOD, and tractography visualizations. The results clearly show enhanced coherence and definition in the white matter pathways after applying HAITCH.

## 5 Discussion

Fetal dMRI faces unique challenges due to inherent motion and susceptibility-induced distortions. These factors lead to scattered data in theq-space domain and result in inconsistencies within and across volumes in terms of diffusion encoding direction and the corresponding diffusion-weighted contrast. Additionally, fetal motion and maternal breathing cause significant fluctuations in the B0 inhomogeneity during scans and render static field map correction techniques ineffective. These limitations necessitate dynamic field map estimation and multi-dimensionalq-space reconstruction for reliable analysis of the fetal brain microstructure. HAITCH addresses these challenges by incorporating several strategies. First, the recommended multi-shell HARDI sampling scheme in HAITCH enriches the information content of the acquired data while enhancing its tolerance to fetal motion. Second, HAITCH leverages a dual-echo EPI sequence for dynamic field map estimation. Classic static field map correction shows inaccurate results due to motion occurring between the two unweighted (b = 0) scans. Third, HAITCH builds on a data-driven representation that spans both the angular and the radialq-space domain, using SH alongside generalized Laguerre polynomials.

Motion generally occurs in three types, each with different impacts on the acquired data: (i) intra-slice motion, which happens within the same slice across multiple volumes, (ii) inter-volume motion, which occurs between volumes, and (iii) inter-slice (intra-volume) motion, which takes place within a single volume but between different slices. In our framework, intra-slice motion is treated as an outlier and rejected through a three-tier weighting process that includes the ModifiedZ-score, Gaussian Mixture Model (GMM), and voxel-wise weighting. The signal of that slice is recovered by other slices that have surrounding gradient directions as described above. Inter-volume motion is corrected using volume-level registration. Fetal diffusion MRI, particularly with multi-shell acquisitions, presents unique challenges due to the inherently low SNR of many acquired volumes (e.g., 56 out of 95 volumes at 900 s/mm^2^). Traditional SVR tools often struggle with low-SNR data and are less effective in this context, which is why HAITCH adopts volume-level registration. This approach has proven to be effective across hundreds of processed datasets.

Inter-slice (intra-volume) motion is corrected through the iterative reconstruction process where the weights of each slice are dynamically refined alongside the motion parameters using convex optimization during the computation of SHORE coefficients. For instance, consider a slicei,jfrom volumeithat is affected by intra-slice motion. Initially, the weight of this slice (based on the Modified Z-score) may remain high, as it does not exhibit obvious signal dropouts. However, as the iterations progress, the weight (now based on the Gaussian Mixture Model (GMM)) will iteratively decrease as individual voxels within the slice are reweighted according to their contribution to the SHORE coefficient computation. In the final iteration of HAITCH, we incorporate voxel-wise weighting to account for small-scale motion effects and other factors (e.g., spin-history effects, physiological motion, fat-shift artifacts, and leakage from accelerated imaging). By assuming that a decent portion of the data remains unaffected by motion, this adaptive data-driven solution helps mitigate those types of motion and other artifacts, leading to an accurate reconstruction of the fetal dMRI data.

The reconstructed fetal dMRI data obtained from HAITCH allows the fitting of various diffusion models, which paves the way for subsequent processing steps and enables a deeper understanding of tissue microstructure and white matter connectivity within the developing brain. As demonstrated inFigures 9and10, the HAITCH correction significantly improves NODDI parameter estimates and streamline tractography by mitigating motion and distortion artifacts. However, it is important to note several challenges specific to fetal imaging that limit the quality of NODDI microstructural maps and tractography. First, limited diffusion sensitivity is a constraint in fetal dMRI. Typically, fetal dMRI is acquired at relatively low b-values (400 and 900 s/mm^2^in our study), which may not capture the full range of microstructural properties required for models like NODDI. Higher b-values (e.g., 2000–3000 s/mm^2^) are often needed to differentiate between intracellular and extracellular compartments. However, achieving such b-values in fetal imaging is challenging due to the low SNR. Second, reduced contrast at low b-values affects the quality of diffusion data. At lower b-values, the diffusion signal attenuation is less pronounced, which reduces the contrast between different tissue types (e.g., gray matter vs. white matter). This limits the ability of the NODDI model to accurately map microstructural features, as the necessary signal differences between compartments are not as detectable. Given these limitations, NODDI and other advanced microstructural models, which are typically designed for multi-shell data with higher b-values, may not provide results comparable to those seen in adolescents and adults data. In such cases, simpler and non-microstructure diffusion models like Diffusion Tensor Imaging (DTI) may yield more reliable results, as illustrated inFigure 10.

HAITCH is the first and the only publicly available tool, to date, to process multi-shell and/or multi-echo fetal dMRI data. While validating the accuracy of HAITCH, it was not feasible to quantitatively compare it with the existing methods due to their limitations - limitations that HAITCH sought to address. The SVRTK (Deprez et al., 2020) toolkit includes modules for fetal dMRI processing, but is restricted to single-shell data and cannot readily process multi-shell or multi-echo dMRI data. The Spherical Harmonics And Radial Decomposition (SHARD) (Christiaens et al., 2021), which was originally developed for neonatal dMRI data, requires an interleaved phase encoding scheme, which is based on a specific sequence (described inBhushan et al., 2014). SHARD utilizes an invariant B0 field map and neglects dynamic field variations. An extended version of SHARD for fetal dMRI, explained byCordero-Grande et al. (2018), utilizes the spin and field echo (SAFE) sequence for phase-based dynamic B0 estimation followingDymerska et al. (2018). While promising, this method relies on MRI sequences that may not be accessible to many researchers, and involves complex phase subtraction, which can be challenging.Dymerska et al. (2018)reported limited accuracy of phase-based B0 estimation in the presence of motion exceeding 8.1 degrees, which may not be sufficient for all fetal MRI applications.Hutter et al. (2018)proposed a slice-level diffusion encoding and interleaved double spin-echo sequence for distortion and motion correction. We did not have access to this sequence and did not re-implement it on our scanner platform. Future work warrants a detailed comparison between various sequences and their effectiveness in the presence of fetal motion, geometric artifacts, and intensity distortions that vary by fetal and maternal motion.

The information gained by fetal diffusion MRI cannot be obtained by any other imaging modality. Therefore, there has been significant interest in studying the development of the fetal brain microstructure and structural connectivity using dMRI. Early*ex-vivo*studies (Huang & Vasung, 2014;Huang et al., 2009;Takahashi et al., 2012,^2014^;Vasung et al., 2010,^2017^) have shed light on the capacity of dMRI to study the microstructure of the fetal brain. These studies were compared to histology and have also been used to indirectly validate*in-vivo*studies, for example,Kebiri et al. (2024). However, postmortem fetal brain samples are scarce, and may not be available in sufficient numbers to study any specific disorder.*In-vivo*fetal dMRI is challenging due to fetal motion, geometric distortion, and the extremely low SNR available from the small fetal brain anatomy. Despite these impediments, significant progress has been made in the field: various motion-robust fetal dMRI techniques have been proposed (Deprez et al., 2020;Fogtmann et al., 2013;Marami et al., 2017;Oubel et al., 2012), atlases have been built and released (Calixto, Soldatelli, et al., 2024;Calixto, Taymourtash, et al., 2024;Chen et al., 2022;Khan et al., 2019), and several studies characterized normal and abnormal development of the fetal brain based on dMRI (Calixto, Machado-Rivas, et al., 2024;Calixto et al., 2023;Jaimes et al., 2020;Jakab, 2019;Machado-Rivas et al., 2021).

Almost all of these works, however, were based on the diffusion tensor model, which is known to have significant limitations in depicting complex fiber structures and connections. Advanced diffusion models that are needed to characterize complex brain connections and study subtle differences between groups of normal and abnormal fetuses, require multi-shell high-angular resolution dMRI, which cannot be processed reliably with any of the existing tool for fetal dMRI processing. By providing an open-source multi-stage framework for multi-shell high-angular resolution fetal dMRI, HAITCH fills in this critical gap.

## 6 Conclusion

We have developed HAITCH, a novel framework and an open-source public toolkit for acquiring and reconstructing high-quality fetal diffusion-weighted MRI. HAITCH utilizes a dual-echo EPI sequence which enables the dynamic field map estimation and reduces motion-related distortions. Our framework employs an advanced scheme that enriches the informational depth of the data and improves its motion tolerance. For motion correction and reconstruction, HAITCH leverages neighborhood directional information and a sophisticated data-driven*model-free*representation in conjunction with slice weighting and registration, effectively addressing signal dropouts and scattered data. Rigorous validation experiments on fetal dMRI scans have demonstrated HAITCH’s ability to significantly improve the integrity and reliability of fetal brain diffusion MRI data, paving the way for more accurate analyses of fetal brain development. The enhanced data quality facilitated by HAITCH has the potential to unlock new insights into the complex processes of brain development*in-utero*.

## Data Availability

The code is publicly available inhttps://github.com/FEDIToolboxandhttps://github.com/IntelligentImaging/HAITCH.
